# Perceptions and knowledge of antipsychotics among mental health professionals and patients

**DOI:** 10.1192/pb.bp.116.055483

**Published:** 2017-10

**Authors:** Lindah Cahling, Anders Berntsson, Gabriella Bröms, Lars Öhrmalm

**Affiliations:** 1 PRIMA, Stockholm, Sweden; 2 Karolinska Instituted Stockholm, Sweden

## Abstract

**Aims and method** To assess the patients' most influential concerns regarding long-acting injectable antipsychotics (LAIs) and mental health professionals' preconceptions about these concerns. For both groups, to assess the level of knowledge about LAIs. This cross-sectional study used semi-structured interviews of patients with schizophrenia or schizoaffective disorder (*n* = 164), nurses (*n* = 43) and physicians (*n* = 20).

**Results** The mental health professionals overestimated many of the patients' fears of LAIs, and the expressed fears exceeded the actual experiences of patients already on LAIs. Acceptance to switch to LAIs was associated with shorter time from diagnosis. Nurses and patients disclosed limited knowledge of antipsychotics.

**Clinical implications** Physicians and nurses should aim to identify the individual patient's concerns about LAIs in the discussion about choice of antipsychotic treatment early in the course of illness.

Adherence to antipsychotic treatment is a major challenge and an important predictor of the outcome in patients with schizophrenia and schizoaffective disorder.^1^ The risks of relapse and admission to hospital increase immediately after discontinuation, even with small treatment gaps.^2–5^ With oral antipsychotics, such gaps often go undetected until relapse – an issue that can be overcome by use of long-acting injectable antipsychotics (LAIs).^5^ There is growing evidence that, compared with oral treatment, LAIs reduce the risk of discontinuation, relapse and hospital admission.^6^ Furthermore, owing to superior pharmacokinetics, the use of LAIs is considered to increase the likelihood of finding the lowest effective dose, which subsequently reduces the risk of side-effects.^7,8^

Despite the identified advantages, LAIs are not used as widely as might be expected. The prescription frequency varies greatly between countries,^9^ indicating that factors other than the patient's attitude influence the utilisation rate. While patients' attitudes towards LAIs become more positive with increased knowledge and experience of the treatment,^10^ clinicians often overestimate patients' resistance against LAIs, anticipating that they will be concerned about the injection procedure.^10–12^ This impedes so-called shared decision-making, an approach with the potential to increase adherence.^13^ Patients are frequently excluded from the discussion about the choice of antipsychotic formulation,^14^ and one reason may be resistance arising from mental health professionals' preconceptions.

We aimed to investigate the specific concerns that affect patients' perceptions of LAIs, and to what extent mental health professionals' preconceptions agree with these perceptions. Furthermore, we aimed to identify knowledge gaps about antipsychotic formulations among both patients and mental health professionals.

## Method

### Design

We conducted a cross-sectional study of mental health professionals' and patients' perceptions and knowledge regarding antipsychotic treatment in a psychiatry catchment area in Stockholm, Sweden, operated by PRIMA Adult Psychiatry. Data were collected in semi-structured interviews performed by a research nurse (L.C.) at the participant's home clinic between January and October 2015. The participants were enrolled upon giving written informed consent. The study was approved by the regional ethical review board in Stockholm (ref. 2015/47-31).

### Participants and setting

We studied three categories of participants: (a) patients on LAIs; (b) patients on oral treatment; (c) mental health professionals, including physicians and nurses.

We identified all patients with schizophrenia or schizoaffective disorder. Patients with no medical treatment or with previous but discontinued LAIs, as well as patients who had been on LAIs >5 years were excluded. Other exclusion criteria were language barriers (i.e. need of interpreter in consultations), cognitive impairment and severe autism spectrum disorders. We collected information on patients' age, gender, years with diagnosis, marital status, number of children, highest achieved academic degree and occupation. The patients on oral medication were block randomised on diagnosis (schizophrenia/schizoaffective disorder), gender and age to two separate arms. The first group was included in this study to represent those on oral medication, while the second group did not participate and will act as controls in a future intervention study.

All physicians and nurses working in the psychosis sector of PRIMA were asked to participate. We recorded age, gender and extent of experience within the psychiatric field.

### Questionnaires

We conducted semi-structured interviews based on different questionnaires specific to each participant category and designed for the present study. Participants graded their potential concerns with a mark on a continuous 100 mm scale ranging from 0 (‘Does not affect at all’) to 100 (‘Decisive to decline LAIs’). Potential concerns included in the questionnaire were pain at administration, possible observation time of 3h, embarrassment at administration, restricted autonomy, feeling of being controlled, being obliged to show up at the clinic regularly, lack of ability to decide when medication is administered, and stigmatisation. The questions were designed to address the participant appropriately, for example, mental health professionals: ‘To what extent do you think fear of pain affects the patients' perception?’; patients on oral medication: ‘To what extent does fear of pain affect your perception?’; and patients on LAIs: ‘To what extent does pain affect your perception?’. The investigated concerns were predefined based on a literature review and clinical experience.^10,15,16^

Questions about knowledge of the differences between LAIs and oral treatment regarding achieved plasma concentration, side-effects and risk of readmission to hospital were identical for all participants and included pre-specified nominal options. In questions regarding clinical approach among mental health professionals, participants were asked to state items freely Physicians stated their most common reasons for prescribing LAIs, and their strategies to encourage patients to consider LAIs. Furthermore, they were asked to speculate what the patients' key reasons for accepting LAIs are. These answers were assessed using thematic analysis after the study was complete.

Nurses were asked whether they tried to influence patients' and physicians' choice of formulation (yes/no). Patients on oral medication were asked whether they had previously been offered LAIs and whether they knew of the features of being on LAIs. Finally, at the end of the interview, all patients were asked about their perspective on switching formulation (positive/negative).

### Statistical analysis

Anonymised data were analysed using Prism 5.03 for Windows. Sample comparisons were made using Fisher's exact test for categorical variables (gender, positive/negative towards switching to LAI). The Mann–Whitney [*U*-test or Wilcoxon matched-pairs test was used for continuous variables (age, illness duration, mental health professionals' experience and questionnaire responses on a 100 mm scale), where appropriate.

## Results

### Inclusion and exclusion of study participants

**Fig. 1:**
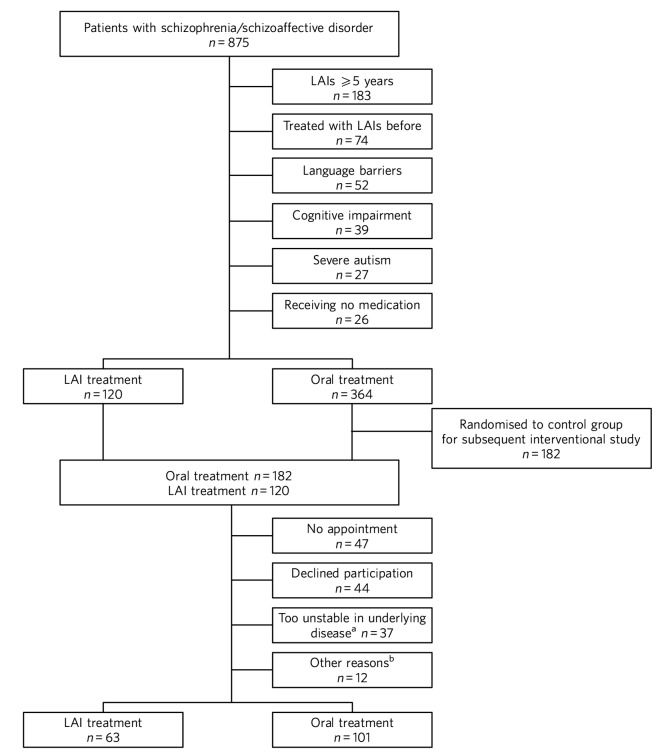
Flow chart of inclusion in the study. LAI, long-acting injectable antipsychotic. a. As assessed at the time of interview, b. No longer a patient at the clinic, changed formulation before interview, deceased and cognitive impairment.

We identified 875 patients in the catchment area with a diagnosis of either schizophrenia or schizoaffective disorder; 341 patients were currently being treated with LAIs (39%). Of the 875 patients, 302 met our inclusion criteria. Finally, 101 patients on oral treatment and 63 on LAIs participated in the study. The reasons for not participating are presented in Fig. 1. All 21 physicians and 46 nurses working in the psychosis sector of PRIMA were asked to participate; 1 physician and 3 nurses declined owing to lack of time.

### Participant characteristics

There were no statistical differences regarding characteristics between patients on LAIs and patients on oral medication, except that patients on oral medication were more likely to have achieved a higher academic degree (Table 1). The median age of the 20 physicians was 47 years (range 34–69) and 45% were women. The median number of completed years in the psychiatric field was 6.5 years (range 0–20). The 43 nurses had a median age of 51 years (range 27–67) and 81% were women. The median length of experience in the psychiatric field was 12 years (range 0–36), and 51% were specialists in psychiatric care.

**Table 1:** Characteristics of interviewed patients

Characteristics	Patients on oral treatment (*n* = 101)	Patients on LAIs (*n* = 63)	*P*
Females, *n* (%)	46 (46)	26 (41)	n.s.

Age, years: median (range)	50 (21–84)	51 (24–74)	n.s.

Diagnosis, *n* (%)			
Schizophrenia	71 (70)	41 (65)	n.s.
Schizoaffective disorder	30 (30)	22 (35)	n.s.

Duration of illness, years: median (range)	21 (1–55)	18 (1–45)	n.s.

Highest education, *n* (%)			
Elementary school	21 (21)	24 (38)	0.020
High school	49 (49)	31 (49)	n.s.
University	31 (31)	8 (13)	0.0086

Employed, *n* (%)	19 (19)	7 (11)	n.s.

Marital status, *n* (%)			
Single	71 (70)	53 (84)	n.s.
Living independently	8 (8)	2 (3)	n.s.
Married/cohabiting	22 (22)	8 (13)	n.s.

Underage children living at home, *n* (%)	8 (8)	3 (5)	n.s.

LAIs, long-acting injectable antipsychotics; n.s., not significant.

### Perceptions of LAI antipsychotics

Comparing patients on oral antipsychotics *v*. patients on LAIs, fears exceeded the actual experiences for all factors examined (Table 2, online Fig. DS1), but fear was only statistically significant for the concerns of being tied to the clinic (62 *v*. 28, *P* = 0.018) and loss of decision-making regarding when to take the medicine (45 *v*. 8, *P* = 0.001). Overall, patients' results tended to be polarised to either end of the 100 mm scale whereas the mental health professionals' results were more centred in their distribution.

**Table 2:** Estimated and actual fears as well as experienced factors affecting the decision to decline long-acting injectable antipsychotics (LAIs)

Factors	Mental health professionals (*n* = 63)	Patients on oral treatment (*n* = 101)	*P*	Patients on LAIs (*n* = 63)	*P*^a^
Pain at the injection site	50.5 (11–95)	28 (0–100)	0.001	12 (0–100)	0.21

Being regularly tied to a clinic	49 (5–98)	62 (0–100)	0.24	28 (0–99)	0.018

Observation time at the clinic after one certain type of LAI^b^	64 (4–98)	89 (0–100)	<0.0001	67 (3–98)	0.23

Embarrassment of having an injection	41 (1–93)	12 (0–98)	<0.0001	7 (0–100)	0.11

Reduction in autonomy	62 (5–93)	30 (0–98)	0.0025	10 (0–100)	0.18

Loss of ability to decide when to take the medication	56 (4–95)	45 (0–100)	0.13	8 (0–98)	0.001

Feeling of being controlled	56 (3–94)	25 (0–100)	0.013	13 (0–100)	0.58

Perceptions of stigma of being on LAI	51 (4–88)	17 (0–100)	0.0004	11 (0–100)	0.63

The questions were presented orally and adapted based on the participant category, i.e. mental health professionals, patients on oral treatment and patients on LAIs.

a. Patients on oral treatment *v*. patients on LAIs.

b. Only the 7 patients on long-acting injectable olanzapine who had experienced a 3 h observation time were included.

Patients on LAIs were asked to recall their fears before switching from oral treatment. They graded their recalled fears higher than the actual experiences regarding all factors except for observation time (online Table DS1). The differences were small, but reached statistical significance for pain (24 *v*. 12, *P* < 0.0001), embarrassment (9 *v*. 7, *P* = 0.0006), reduction in autonomy (13 *v*. 10, *P* = 0.0027) and loss of ability to decide when to take the medicine (14 *v*. 8, *P* = 0.019). Finally, there were no statistically significant differences between the graded fears of patients on oral treatment *v*. recalled fears in patients on LAIs (data not shown).

Mental health professionals overestimated the concerns of orally treated patients regarding feared pain (51 *v*. 28, *P* = 0.001), embarrassment (41 *v*. 12, *P* < 0.0001), reduction in autonomy (62 *v*. 30, *P* = 0.0025), feeling of being controlled (56 *v*. 25, *P* = 0.013), and stigma (51 *v*. 17, *P* = 0.0004; Table 2, online Fig. DS1). Conversely, they underestimated the patients' concerns regarding the 3h observation time required after injection of LAI olanzapine (64 *v*. 89, *P* < 0.0001).

### Knowledge of oral and LAI antipsychotics

All physicians (100%) claimed that LAIs are associated with a more stable plasma concentration than oral treatment (Table 3). For nurses, patients on oral treatment and patients on LAIs, the corresponding proportions were 56%, 16% and 22%, respectively.

**Table 3:** Mental health professionals' and patients' knowledge about oral *v.* long-acting injectable antipsychotics (LAIs) regarding plasma concentration, side-effects and frequency of readmission to hospital

Topic	Physicians (*n* = 20)	Nurses (*n* = 43)	Patients on oral treatment (*n* = 101)	Patients on LAIs (*n* = 63)
Plasma concentration, *n* (%)				
Lower/more stable with LAIs	20 (100)	24 (56)	16 (16)	14 (22)
Equal	0 (0)	11 (26)	23 (23)	20 (32)
Lower/more stable with oral	0 (0)	4 (9)	41 (41)	20 (32)
Don't know	0 (0)	4 (9)	21 (21)	9 (14)

Side-effects, *n* (%)				
Less with LAIs	15 (75)	12 (28)	18 (18)	27 (43)
Equal	3 (15)	17 (40)	25 (25)	19 (30)
Less with oral	1 (5)	11 (26)	45 (45)	11 (17)
Don't know	1 (5)	3 (7)	13 (13)	6 (10)

Risk of rehospitalisation, *n* (%)				
Less with LAIs	19 (95)	37 (86)	21 (21)	23 (36)
Equal	0 (0)	3 (7)	40 (40)	20 (32)
Less with oral	1 (5)	2 (5)	15 (15)	5 (8)
Don't know	0 (0)	1 (2)	25 (25)	15 (24)

Eligible answers were presented as pre-specified nominal options.

Of physicians, 90% stated that LAIs are superior or equal to oral treatment concerning side-effects. For nurses, patients on oral treatment and patients on LAIs, the corresponding proportions were 68%, 43% and 73%, respectively.

All physicians but one (95%) and 86% of nurses claimed that LAIs reduce the risk of readmission to hospital, while 21% of patients with oral treatment and 36% of patients on LAIs claimed LAIs to be superior in this matter.

### Clinical approach to LAIs among mental health professionals

Poor adherence, limited insight and multiple relapses were the most common reasons for prescribing LAIs, mentioned by 80% of physicians. However, one-fourth considered LAIs an option even early in the disease course. Their strategies to encourage patients to consider LAIs were to inform them about the advantages of the formulation (65%) and about the risks and consequences of treatment discontinuation (40%). Exploring patients' fears was a strategy mentioned by 20% of physicians.

Half of physicians believed that not having to remember to take pills was the key reason for patients to accept LAIs. Other factors mentioned were good insight (40%) and that LAIs are associated with lower frequency of relapse (20%).

Of nurses, 31 (72%) replied that they actively tried to influence the patients' attitude towards one or the other formulation, and 29 (67%) actively tried to influence the physician's decision.

### Patients' perspective on switching

Almost half of the patients on oral treatment (41%) declared that they had little or no knowledge of LAIs. At the end of the interview, they were asked whether they would switch to LAIs if offered by their treating physician. While 78 (77%) said no and three (3%) could not decide, 20 (20%) declared that they would agree to switch if offered such an option. The patients willing to switch had fewer years since diagnosis than those who were reluctant (12 *v*. 24, *P* = 0.0013; online Fig. DS2). Furthermore, the proportion of women was higher in the positive group (75% *v*. 44%, odds ratio (OR) = 3.9, *P* = 0.023). They considered pain (7 *v*. 40, *P* = 0.020), being tied to the clinic (26 *v*. 70, *P* = 0.017), reduction in autonomy (9 *v*. 30, *P* = 0.034) and stigma (6 *v*. 27, *P* = 0.035) to be less important issues than did the patients who were reluctant to switch to LAIs.

A total of 21 (33%) patients on LAIs would switch to oral treatment if they were offered it, 1 (1.6%) could not decide and 41 (65%) preferred to continue with LAIs. There were no statistically significant differences between patients who were positive *v*. patients who were negative about switching formulation with regard to age, number of years with diagnosis or gender. Those who opted to stay on LAIs were less concerned with the lack of autonomy (7 *v*. 40, *P* = 0.015) and the feeling of being controlled (9 *v*. 50, *P* = 0.0011). They also gave more correct answers regarding differences in side-effects between oral formulations and LAIs (85% *v*. 52%, OR = 5.3, *P* = 0.012).

## Discussion

In this study, we found that patients' concerns with LAIs were minor except when considering observation time and being tied to the clinic, and that there was a mismatch in the assessment of specific concerns between the patients and the mental health professionals. We identified important knowledge gaps among patients and nurses. As many as one-fifth of the patients on oral medications were willing to switch to LAIs; these potential switchers were more recently diagnosed than those who were reluctant.

The patients on oral treatment were most concerned about observation time post-injection and about being tied to the clinic when asked about LAIs. This indicates that they valued their time and that practical issues surpassed in significance emotional ones such as stigma, a feeling of being controlled and embarrassment. All fears expressed by patients on oral treatment exceeded the actual experiences of patients on LAIs. This could be a result of selection bias, in that patients on LAIs were less concerned even before accepting LAI treatment. However, since patients on LAIs were speaking from experience, this difference may also reflect that these issues had a lower impact than expected once the patients had been started on LAIs. That the recalled concerns pre-LAIs were similar to the levels of concern among those still on oral treatment also supports this hypothesis.

Mental health professionals tended to answer questions by placing the indicator centrally on the 100 mm scale, which may reflect uncertainty as they were just estimating the patients' experiences. The patients' answers, on the other hand, were polarised, indicating that their opinions were more set. Patients also graded some factors distinctly low and others distinctly high. In light of this, physicians should be encouraged to learn more about the individual patient's concerns. Only 20% of physicians reported that they used this strategy when discussing treatment regimens.

According to previous studies, physicians' knowledge regarding antipsychotic formulations varies.^16,17^ Physicians in the current study showed very good knowledge. However, a significant proportion of the interviewed nurses had knowledge gaps concerning some of the advantages of LAIs. This could have a negative impact on the patient's attitude towards LAIs, especially as the majority of nurses claimed that they actively tried to influence both doctors and patients in the discussion on treatment choices. Patients already on LAIs had significantly better knowledge about the reduced side-effects with LAIs than patients on oral treatment. This most likely reflects their own experiences. It could also be an effect of information provided by mental health professionals – information many patients on oral treatment reported as lacking. This is of concern, as we know that patients' attitudes towards LAIs are likely to become more positive with increased knowledge and experience of the treatment.^10^ The physicians' observed reluctance to bring up the topic may be due to their anticipation that the patients are unlikely to accept the offered LAI. However, keeping the patients uninformed makes shared decision-making impossible.^15^

The majority of the patients on LAIs chose to keep this formulation and as many as 20% of the patients on oral treatment were willing to use LAIs. This is in line with a previous study in which 16% were positive towards a formulation switch.^14^ This also supports the hypothesis that the use of LAIs could be limited by factors other than rejection by the patients.^12^ Some physicians claimed that they offered LAIs early in the disease course, but their most common reasons for prescribing LAIs were poor adherence to oral medication and recurring relapses. Previous studies also report non-adherence^16,17^ and multiple relapses^17^ as key criteria for prescribing LAIs. This may be unfortunate as longer illness duration was associated with being reluctant to switch. Instead, this motivates a discussion of LAIs early on in the course of illness, especially as there is cumulative evidence that the use of LAIs as early as after the first admission to hospital decreases the risk of treatment discontinuation, relapse and readmission.^4,5,18^

### Limitations

Our study has several limitations. Not all patients in the targeted study population were included, and some patients could not be reached or were not present to complete the questionnaire. Some were only scheduled for visits once per calendar year, while the study was limited to 10 months. It is possible that patients were either too ill to present themselves or were stable enough to postpone yearly visits. The patients on LAIs were asked to declare their perceptions prior to starting on LAIs, which introduced recall bias. However, we excluded all patients on LAIs ⩾ 5 years, reducing the effect of this bias. Finally, patients on LAIs are indisputably a selection of patients who have once accepted that formulation. However, the lack of significant differences between the graded fears of patients on oral treatment compared with recalled fears in patients with LAIs may indicate that this selection bias is of minor concern. A strength of this study was that all interviews were performed by the same person (L.C.), securing consistency across interviews.

### Clinical implications

In conclusion, physicians should aim to set aside their own preconceptions and instead make time to identify the individual's specific fears regarding LAIs, preferably early in the course of the illness. In addition, there is room for improvement regarding patients' knowledge of antipsychotic formulations. Adequate education would be of value to strengthen nurses' knowledge about LAIs. Finally, there is room for improvement regarding patients' knowledge of antipsychotic formulations.
